# Microtubule-organizing center polarity and the immunological synapse: protein kinase C and beyond

**DOI:** 10.3389/fimmu.2012.00235

**Published:** 2012-07-31

**Authors:** Morgan Huse

**Affiliations:** Immunology Program, Memorial Sloan-Kettering Cancer Center, New York, NY, USA

**Keywords:** cell polarity, cytoskeleton, lymphocyte, protein kinase C, signal transduction, T cell

## Abstract

Cytoskeletal polarization is crucial for many aspects of immune function, ranging from neutrophil migration to the sampling of gut flora by intestinal dendritic cells. It also plays a key role during lymphocyte cell–cell interactions, the most conspicuous of which is perhaps the immunological synapse (IS) formed between a T cell and an antigen-presenting cell (APC). IS formation is associated with the reorientation of the T cell’s microtubule-organizing center (MTOC) to a position just beneath the cell–cell interface. This cytoskeletal remodeling event aligns secretory organelles inside the T cell with the IS, enabling the directional release of cytokines and cytolytic factors toward the APC. MTOC polarization is therefore crucial for maintaining the specificity of a T cell’s secretory and cytotoxic responses. It has been known for some time that T cell receptor (TCR) stimulation activates the MTOC polarization response. It has been difficult, however, to identify the machinery that couples early TCR signaling to cytoskeletal remodeling. Over the past few years, considerable progress has been made in this area. This review will present an overview of recent advances, touching on both the mechanisms that drive MTOC polarization and the effector responses that require it. Particular attention will be paid to both novel and atypical members of the protein kinase C family, which are now known to play important roles in both the establishment and the maintenance of the polarized state.

Lymphocytes can completely alter their cellular architecture in a matter of minutes in response to cell surface stimulation. This enables them to adapt quickly to multiple disparate tissue environments, which is crucial for effective migration between and within different organ systems. Structural plasticity also plays a key role in promoting and specifying interactions between lymphocytes and other cells. Particularly important among lymphocyte interactions is the immunological synapse (IS), a stereotyped cell–cell contact characterized by the organization of cell surface receptors, adhesion molecules, and signaling proteins into well-defined concentric domains (Dustin et al., 2010). Although the IS was first observed in conjugates between T cells and antigen-presenting cells (APCs), it is clear that natural killer (NK) cells and B cells use similar structures to engage target cells and cells coated with surface-bound antigen, respectively (Dustin and Long, 2010; Harwood and Batista, 2010).

Immunological synapse formation is accompanied by a dramatic change in cell shape. This transformation has been studied most extensively in T cells, which form synapses in response to antigen recognition by the T cell receptor (TCR). T cells search their environment for antigen in a crawling, “hand mirror” configuration that consists of a leading edge followed by a trailing stalk-like projection known as a uropod (Ward and Marelli-Berg, 2009; **Figure 1**). TCR stimulation transforms the leading edge into a radially symmetric lamellipodium that spreads over the surface of the APC, and concomitantly induces collapse of the uropod (Dustin et al., 2010). Within minutes, the extended crawling morphology of the T cell transforms into a more compact shape akin to a sideways cup with its mouth positioned at the IS (Dustin et al., 2010; **Figure 1**).

**FIGURE 1:**
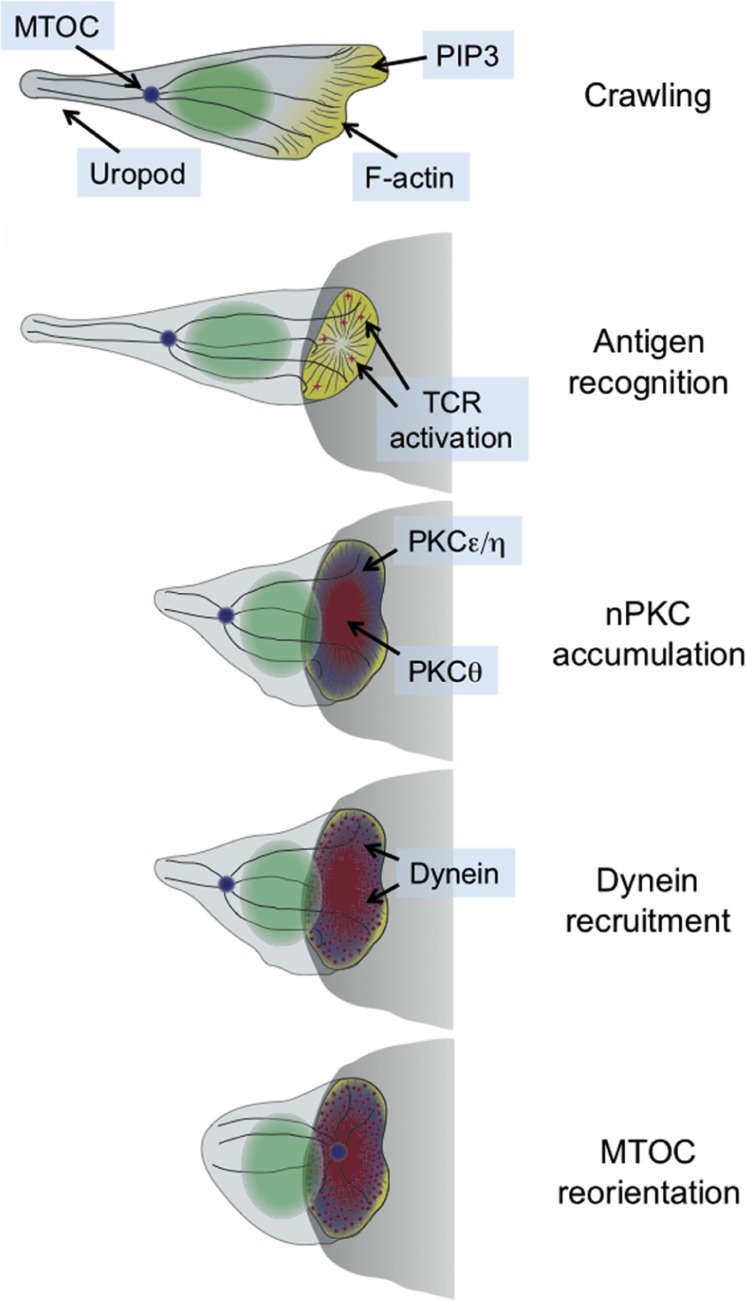
**Schematic diagram showing the transition between migratory and synaptic polarity that occurs upon antigen recognition by T cells**. In general, the entire process takes 2–3 min. The APC is shown as a gray semicircle.

This structural transformation is associated with and facilitated by extensive remodeling of both the actin and microtubule cytoskeletons (Gomez and Billadeau, 2008; Dustin et al., 2010). Actin polymerization drives the radial growth of the synapse, and plays an important role in stabilizing adhesive contacts and other receptor–ligand interactions. The microtubule cytoskeleton, for its part, rotates so as to position the microtubule-organizing center (MTOC, also called the centrosome) just beneath the IS (**Figure 1**). The MTOC carries along with it the Golgi apparatus and other vesicular compartments. Hence, its polarization to the IS aligns much of the cellular machinery involved in protein trafficking with the APC. This enables the directional secretion of proteins and other cargo toward the APC, which is thought to be crucial for maintaining the specificity of T cell responses (Huse et al., 2008). T cells operate in a dense intercellular milieu packed with healthy bystander cells, and yet paradoxically they use secreted factors for a sizable chunk of their effector function. The importance of directional release into the synapse is most obvious in the case of cytotoxic T cells and NK cells, which kill target cells using soluble, cytolytic factors such as perforin and granzyme (Stinchcombe and Griffiths, 2007). It goes without saying that the effects of these agents must be limited to the target cell alone.

Over the past two decades, our knowledge of the signaling pathways associated with TCR activation has improved dramatically. In contrast, our understanding of the molecular mechanisms that drive concomitant cell shape changes and cytoskeletal polarization remains quite poor. In recent years, however, high-resolution imaging approaches have provided investigators with the wherewithal to actually explore the cell biology of T cell activation. This review will focus on what these approaches have taught us about MTOC polarization to the IS, with particular emphasis on the roles played by distinct protein kinase C (PKC) isoforms at various stages during the process.

## MTOC POLARIZATION IS DICTATED BY THE TCR

MTOC reorientation to the IS was first documented three decades ago (Geiger et al., 1982; Kupfer et al., 1983). The process was difficult to study, however, largely because it occurs so quickly (typically within 5 min of receptor stimulation) and because T cells are so small. Early efforts focused on defining the basic requirements for the response, and revealed that it was highly dependent on TCR signaling. In a particularly informative set of studies, Burkhardt and colleagues mixed T cells with target cells expressing either the integrin ligand ICAM-1 or agonist peptide-major histocompatibility complex (pMHC; Sedwick et al., 1999). When T cells contacted both kinds of target cell simultaneously, the MTOC polarized toward the cell expressing pMHC. Conversely, actin accumulated at the interface with the cell expressing ICAM-1. In addition to highlighting the importance of TCR signaling, these results provided perhaps the first indication that the molecular pathways involved in integrin-mediated adhesion were separate from those that guided MTOC polarization. Subsequent studies confirmed the dominant role of TCR signaling by demonstrating that several receptor proximal proteins, including the tyrosine kinases Lck and Zap70 and the scaffolding proteins LAT and Slp76, were required for polarization responses (Lowin-Kropf et al., 1998; Kuhne et al., 2003). These proteins, however, play an important role in nearly every aspect of T cell activation. Hence, it remained unclear precisely how early signals emanating from the TCR are coupled specifically to the MTOC.

## THE IMPORTANCE OF DIACYLGLYCEROL

As mentioned above, the mechanistic analysis of MTOC reorientation to the IS has long been limited by technical constraints imposed by the dynamics of the response and the size of the cells in question. Beyond this, there were certain complicating issues related to the experimental systems used to quantify polarization. MTOC reorientation was typically assessed by live or fixed imaging of T cell–APC conjugates. In this context, observable polarization could only occur after productive contact formation. Hence, molecules or pathways involved in promoting adhesion with the APC would be implicated in MTOC reorientation, even if their effects on the pathway itself were merely secondary.

To circumvent these issues and improve the spatial and temporal resolution of analysis, we developed a single cell polarization assay in which conjugate formation with an APC was replaced by controlled stimulation of the TCR in a micron-sized region of the T cell membrane (Huse et al., 2007). Our approach is based on a photoactivatable pMHC reagent specific for the 5C.C7 TCR. This reagent bears a large, photocleavable group that blocks TCR binding until it is cleaved off with a pulse of ultraviolet (UV) light. Primary 5C.C7 T cells expressing some sort of fluorescent signaling probe (typically proteins linked to GFP or RFP) are attached to coverslips containing this photoactivatable pMHC and imaged by video microscopy. After a short interval to establish a baseline recording, a micron-sized region beneath each cell is UV irradiated, creating a zone of agonist pMHC that is competent to bind to the 5C.C7 TCR. Subsequent intracellular signaling and cytoskeletal responses are monitored over the next 5–10 min using either epifluorescence or total internal reflection fluorescence (TIRF) illumination.

This protocol typically induces reorientation of the MTOC to the irradiated region in less than 3 min (Quann et al., 2009), and we have been using it as an assay to mechanistically dissect the process. This approach has several advantages over more standard T cell–APC conjugate experiments, the most obvious being a substantial improvement in spatial and temporal resolution. Indeed, the combination of TIRF microscopy, which provides high-resolution images of the plasma membrane attached to the glass, and the ability to control when and where the T cell is stimulated, has enabled us to resolve events separated by a few as 5 s. In this manner, we have been able to establish a very precise order of operations leading up to MTOC reorientation (Quann et al., 2009, 2011). In addition, because contact with the glass surface is established prior to TCR stimulation (typically using an antibody against a class I MHC protein expressed by the T cells) it is likely that we have isolated the pathways guiding MTOC polarity, which we can study independently of mechanisms controlling adhesion.

Using this assay, we began to explore the relationship between early TCR signaling and MTOC polarization. We were particularly intrigued by the lipid second messenger diacylglycerol (DAG), which is generated by phospholipase C-γ (PLCγ) downstream of TCR activation. DAG transduces signals by recruiting proteins containing DAG-binding C1 domains to the membrane (Colon-Gonzalez and Kazanietz, 2006). It was known that DAG accumulates in a polarized manner at the IS (Spitaler et al., 2006), and we had found that a PLCγ inhibitor completely blocked MTOC polarization (Quann et al., 2009). Using a C1 domain-containing protein biosensor that translocates to membranes containing DAG, we discovered that MTOC reorientation was invariably preceded 10–15 s by the localized accumulation DAG in the region of TCR stimulation (Quann et al., 2009). This close temporal relationship suggested that DAG served to guide the MTOC to the IS. Indeed, using various perturbation approaches, we were able to show that disrupting the ability of the T cell to maintain a localized DAG accumulation or to respond to such an accumulation blocked MTOC polarization. In addition, using a photoactivatable DAG reagent that cannot engage its targets until it is irradiated with UV light, we demonstrated that the localized generation of DAG alone could induce MTOC reorientation, independent of the TCR.

The importance of localized DAG accumulation for the polarization of the MTOC is strikingly reminiscent of the phosphatidylinositol tris-phosphate (PIP3) based direction-sensing mechanism used by *Dictyostelium* and neutrophils to establish migratory polarity (Devreotes and Janetopoulos, 2003; Ward and Marelli-Berg, 2009). In these cell types, the accumulation of PIP3 promotes formation of a leading edge lamellipodium and is important for effective migration within a chemotactic gradient. This system is well suited for rapid and transient direction sensing because PIP3 is continuously metabolized by lipid phosphatases such as PTEN and SHIP, and therefore must be replenished by new PIP3 production in order to maintain directionality. The dynamic balance between production and metabolism enables cells to respond quickly to positional changes in surface receptor stimulation because these changes necessarily lead to positional changes in lipid second messenger production. The same sort of dynamic balance exists for DAG, whose production by PLC isozymes is offset by DAG kinases (DGKs), which convert DAG to phosphatidic acid (PA; Zhong et al., 2008).

The extent to which PIP3-based direction sensing participates in T cell migration is somewhat controversial (Nombela-Arrieta et al., 2004; Reif et al., 2004; Asperti-Boursin et al., 2007; Liu et al., 2007; Smith et al., 2007; Ward and Marelli-Berg, 2009). Nevertheless, it is intriguing to speculate that T cells employ DAG and PIP3 simultaneously as a way to decouple lamellipodial dynamics from MTOC polarization. The ability to control these processes independently would presumably be important for transitioning between synaptic morphology, where the MTOC and leading edge localize to the same interface, and migratory morphology, in which the MTOC localizes to the uropod, distal to the leading edge.

## A CASCADE OF NOVEL PKCs

The discovery that DAG plays an important role in T cell MTOC polarization immediately suggested that proteins containing C1 domains were involved in the process. Of these, perhaps the most obvious candidates were the PKCs. It had been known for some time that a combination of phorbol esters (e.g., PMA) and Ca^2+^ ionophores (e.g., ionomycin) can largely recapitulate the effects of T cell activation independent of the TCR (Chatila et al., 1989). These reagents directly activate multiple PKCs, strongly implicating this family of proteins in T cell signaling. Consistent with this notion, PKC inhibitors effectively block many TCR-induced responses, including proliferation and the secretion of inflammatory cytokines (Baier and Wagner, 2009). Although few studies had implicated PKCs in the regulation of lymphocyte architecture, they were known to play an important role in cytoskeletal remodeling in adherent cells such as fibroblasts (Larsson, 2006).

The protein kinase C family is typically divided into three subgroups, which can be distinguished by the structure of their N-terminal regulatory regions (Newton, 2010). Conventional PKCs (cPKCs) contain tandem, DAG-binding C1 domains followed by a C2 domain, which recognizes negatively charged phospholipids in a Ca^2^^+^-dependent manner. Novel PKCs (nPKCs), by contrast, contain a C2 domain at their N-termini that cannot bind to phospholipids due to mutations in its Ca^2^^+^ binding sites. The tandem C1 domains that follow have an unusually high affinity for phorbol esters and DAG. Atypical PKCs (aPKCs) lack C2 domains entirely, and contain only one C1 domain that has lost the ability to bind DAG. These differences in domain structure endow each PKC subfamily with distinct regulatory properties: cPKCs require both Ca^2+^ and DAG for their activation, nPKCs require DAG alone, while aPKCs are largely regulated through protein–protein interactions.

Identifying which of these isoforms contribute to MTOC polarization responses was complicated by the fact that most, if not all, PKCs are expressed in T cells. The importance of localized DAG, however, argued against a role for aPKCs, at least during the early phases of the response. Furthermore, we had shown that Ca^2+^ signaling was not required for polarization (Quann et al., 2009), suggesting that cPKCs were not involved. Hence, we chose to focus first on the nPKC subfamily, comprising PKCδ, PKCε, PKCη, and PKCθ. Of these, probably the best studied was PKCθ, which is highly expressed in both developing and mature T cells. T cells lacking PKCθ display marked deficiencies in antigen-induced proliferation, cytokine secretion, and development into the T_H_2 lineage (Sun et al., 2000; Marsland and Kopf, 2008). PKCθ is thought to mediate many of these effects by activating several key transcription factors, including NF-κB, NFAT, and AP-1, which together account for a significant fraction of TCR-dependent gene expression (Manicassamy et al., 2006).

T cell receptor signaling induces the accumulation of PKCθ at the IS (Monks et al., 1997), where it would presumably be well positioned to promote cytoskeletal polarization. Prior to our work, however, it was unknown whether PKCθ actually contributed to this process. Even less was known about the other nPKCs. Indeed, previous studies implied that PKCε and PKCη were not required for any aspect of T cell activation (Monks et al., 1997; Gruber et al., 2005). Hence, we were quite surprised to find that TCR stimulation in both photoactivation experiments and T cell–APC conjugates induced the robust IS recruitment of not only PKCθ, but also PKCε and PKCη (Quann et al., 2011). Notably, PKCδ was not recruited in this manner, consistent with previous reports indicating that it localizes to intracellular granules instead (Ma et al., 2008).

The synaptic accumulation of PKCε, PKCη, and PKCθ preceded reorientation of the MTOC (**Figure 1**), consistent with a role for all three proteins in the process. Indeed, siRNA-mediated suppression of either PKCθ alone or PKCε and PKCη in combination disrupted polarization responses (Quann et al., 2011). These results indicated that all three proteins participate in MTOC reorientation, but that PKCε and PKCη can functionally compensate for each other. In retrospect, redundancy between PKCε and PKCη should not have been particularly surprising, given the high level of sequence identity (60%) between the two proteins. This may explain why PKCε-deficient T cells display no observable TCR activation phenotype (Gruber et al., 2005). A more concrete answer will await the analysis of PKCε/PKCη double knockout mice.

Interestingly, simultaneous siRNA knockdown of PKCε and PKCη also inhibited the recruitment of PKCθ, while knockdown of PKCθ did not affect PKCη accumulation (Quann et al., 2011). Taken together, these results suggested that PKCε and PKCη operate upstream of PKCθ to promote MTOC polarization. Close examination of the recruitment dynamics of all three proteins was consistent with this hypothesis (Quann et al., 2011). PKCε and PKCη arrived at the region of TCR stimulation first, followed by PKCθ ~10 s later, and MTOC reorientation 5–10 s after that. PKCε and PKCη had the same accumulation pattern, which covered a broad region of plasma membrane encompassing the entire IS. By contrast, PKCθ occupied a more restricted zone that was entirely contained within the lamellipodial actin ring at the periphery (**Figure 1**). Whether and how these distinct PKC recruitment patterns contribute to polarization responses remains unclear. It is tempting to speculate, however, that the broad accumulation of PKCε and PKCη controls early polarization steps, while the more confined PKCθ distribution contributes to positional refinement of the MTOC at later stages.

We found that the distinct recruitment patterns of PKCη and PKCθ could be largely recapitulated by constructs containing the tandem C1 domains of each protein (Quann et al., 2011). This is remarkable, given that typical C1 domains are all thought to bind to the same ligand, DAG. What then could explain the differences we observed? *In vitro* studies have demonstrated that PKCε binds to bilayers containing DAG with ~10-fold higher affinity than does PKCθ (Stahelin et al., 2005; Melowic et al., 2007). It is likely that the affinity of PKCη for DAG is similar to that of PKCε, given the close homology between the two proteins. The ability of PKCε and PKCη to bind DAG more tightly than does PKCθ would presumably lead to faster IS recruitment. A higher affinity for DAG could also explain why PKCε and PKCη accumulate in a broader membrane zone than PKCθ, assuming that DAG density declines radially outside of the site of TCR stimulation.

Although differential DAG affinity provides an elegant mechanism for modulating PKC recruitment, other results strongly suggest that there are additional contributing factors. For example, PKCδ recognizes DAG with threefold higher affinity than does PKCθ (Stahelin et al., 2004), and yet PKCδ is not recruited to the IS. This probably has less to do with DAG itself and more to do with the complex protein and lipid environment at the IS in which DAG accumulates. The context within which DAG recognition takes place can have dramatic effects on membrane binding by C1 domains. PKCθ, for example, binds to mixtures of DAG and the charged lipid phosphatidylserine with 28-fold higher affinity than it does to DAG mixtures containing the uncharged phosphatidylglycerol (Melowic et al., 2007). The C1 domains of PKCε have been documented to recognize arachidonic acid and ceramide in addition to DAG (Kashiwagi et al., 2002), and various C1 domains engage in protein–protein interactions in the appropriate environments (Colon-Gonzalez and Kazanietz, 2006).

Hence, it seems reasonable to hypothesize that there are either lipid- or protein-based “contextual factors” that contribute in a combinatorial manner to the accumulation of PKCs within the IS. Although the identity of these molecules remains unknown, the potential to modulate C1 domain localization independent of DAG has important implications for the continued use of C1 domains as DAG “biosensors.” Constructs derived from different PKC isoforms as well as protein kinase D are now widely used to monitor *in situ* DAG production in multiple experimental systems. Although we have learned and will continue to learn much from this approach, it is important to keep in mind moving forward that different C1 domains have been evolutionarily tuned to recognize DAG within distinct lipid and protein environments. Therefore, care must be taken when imaging C1 domain constructs, as changes in localization could reflect either a change in DAG density or a change in other contextual factors.

## MOVING THE MTOC WITH MOLECULAR MOTORS

Our mechanistic understanding of MTOC polarization in T cells took a significant step forward when it was discovered that the microtubule motor protein dynein is recruited to the IS (Combs et al., 2006). This immediately suggested that dynein, once anchored at the IS, might reorient the MTOC by pulling on the microtubules that radiate from it. This was an attractive hypothesis because microtubules emerge from the MTOC with their minus ends inward and their plus ends outward, effectively matching the polarity of the dynein motor, which moves from plus end to minus end. Indeed, subsequent siRNA experiments demonstrated that knockdown of dynein impaired MTOC reorientation in Jurkat T cells (Martin-Cofreces et al., 2008).

Dynein is a large, multisubunit protein that contains two copies each of a heavy chain and several accessory light chains (Kardon and Vale, 2009). The heavy chain contains the motor and microtubule binding domains, while the light and intermediate chains provide structural integrity and mediate interactions with accessory factors. The most important of these accessory factors is dynactin, a multisubunit complex roughly equivalent in size to dynein, which enhances dynein processivity and controls its localization to distinct subcellular compartments. Dynein is required for the trafficking of multiple distinct cargos, ranging from proteins such as β-catenin and rhodopsin to organelles like the Golgi apparatus (Vallee et al., 2004; Kardon and Vale, 2009). Although some cargo interactions are mediated by dynein itself, most appear to require dynactin, which plays the role of a large, multifunctional adaptor.

Precisely how the dynein–dynactin complex is recruited to the IS remains unclear. It was initially proposed that dynein associates directly with the TCR signaling machinery by binding to ADAP, a scaffolding protein that interacts with Slp76. In Jurkat cells, ADAP was found to accumulate at the IS, and siRNA-mediated suppression of ADAP inhibited MTOC reorientation (Combs et al., 2006). These results could not be extended to primary T cells, however, raising doubts as to the physiological relevance of this recruitment pathway. TCR photoactivation experiments have revealed that DAG accumulation precedes dynein recruitment by ~10 s, and that DAG and dynein occupy essentially the same region of membrane prior to MTOC reorientation (Quann et al., 2009). This suggests that DAG might recruit dynein either directly or via some DAG-binding adaptor. Although there is little evidence for this at present, dynein and dynactin are very large, and it is not implausible that one of the less well-studied subunits could indeed be regulated by DAG or a related lipid.

While it is generally thought that dynein–dynactin is necessary for MTOC reorientation in T cells, recent work has cast doubt on the idea that it is entirely sufficient. In primary T cells, polarization responses toward supported lipid bilayers containing cognate pMHC were unaffected by siRNA knockdown of dynein or by the small molecule dynein inhibitor EHNA (Hashimoto-Tane et al., 2011). Although these results imply that dynein-independent pathways exist for MTOC reorientation, the precise identity of these pathways remains to be seen. They are unlikely to involve plus end directed microtubule motors, as overexpression of the dynein recruitment factor RILP (see below), which blocks plus end directed movement, had no effect on MTOC reorientation (Stinchcombe et al., 2006). This leaves the cortical actin cytoskeleton, which interfaces with microtubules just beneath the plasma membrane. In the mature IS, microtubules radiating from the MTOC have been documented to intersect with the actin ring that forms at the periphery of the contact (Kuhn and Poenie, 2002). It has been proposed that the formation of this actin ring applies tension to associated microtubules, thereby dragging the MTOC into close apposition with the synaptic membrane (Stinchcombe et al., 2006). In this manner, the force generated by actin polymerization in the radial lamellipodium could be utilized for MTOC reorientation. Although this hypothesis has not been tested directly, it is interesting to note that several proteins involved in coupling the microtubule cytoskeleton with cortical actin, including the scaffolding molecule IQGAP and the Diaphanous formins, have also been implicated in MTOC reorientation (Gomez et al., 2007). It is possible that these proteins promote polarization responses by establishing and maintaining force-bearing contacts between microtubules and actin.

The collaboration between microtubule- and actin-based remodeling during MTOC polarization is best understood in fibroblasts, which position the MTOC in front of the nucleus during cell migration. In this system, dynein functions to hold the MTOC immobile, while retrograde actin flow drives the nucleus behind it (Gomes et al., 2005). These actin dynamics are, in turn, controlled by myotonic dystrophy kinase-related Cdc42 binding kinase (MRCK) and non-muscle myosin II (Gomes et al., 2005). It is not known whether and to what extent this actin-based pathway contributes to the T cell response, which is much faster and less stable than polarization in fibroblasts. Notably, our photoactivation experiments, which drive MTOC reorientation to a fixed region in space, induce robust MTOC movement, often with minimal net nuclear motion (X. Liu and M. Huse, unpublished results). This is the opposite of what is seen in fibroblasts, suggesting that components of actin-based motility, if they do promote MTOC polarization in T cells, do so via a somewhat distinct mechanism.

## STABILIZATION OF THE POLARIZED STATE BY aPKCs

In astrocytes, fibroblasts, and epithelial cells, aPKCs such as PKCζ and PKCι play a central role in the acquisition and maintenance of polarity (Etienne-Manneville and Hall, 2003; Li and Gundersen, 2008). aPKCs operate in this context as part of a complex that also contains the adaptor molecules PAR3 and PAR6 (for partitioning defective). PAR proteins were initially isolated in a screen for factors regulating embryonic polarity, and they are crucial for a variety of processes ranging from asymmetric cell division to the establishment of an apical–basal axis in epithelial cells (Nelson, 2003; Nance and Zallen, 2011). Accumulation of the PAR3–PAR6–aPKC complex in one plasma membrane domain is often accompanied by the accumulation of a distinct complex containing the proteins Scribble and Discs-large (Dlg) in a reciprocal domain. The PAR and Scribble–Dlg complexes mutually antagonize each other’s assembly, effectively enhancing and stabilizing the polarized state (Nelson, 2003).

**FIGURE 2:**
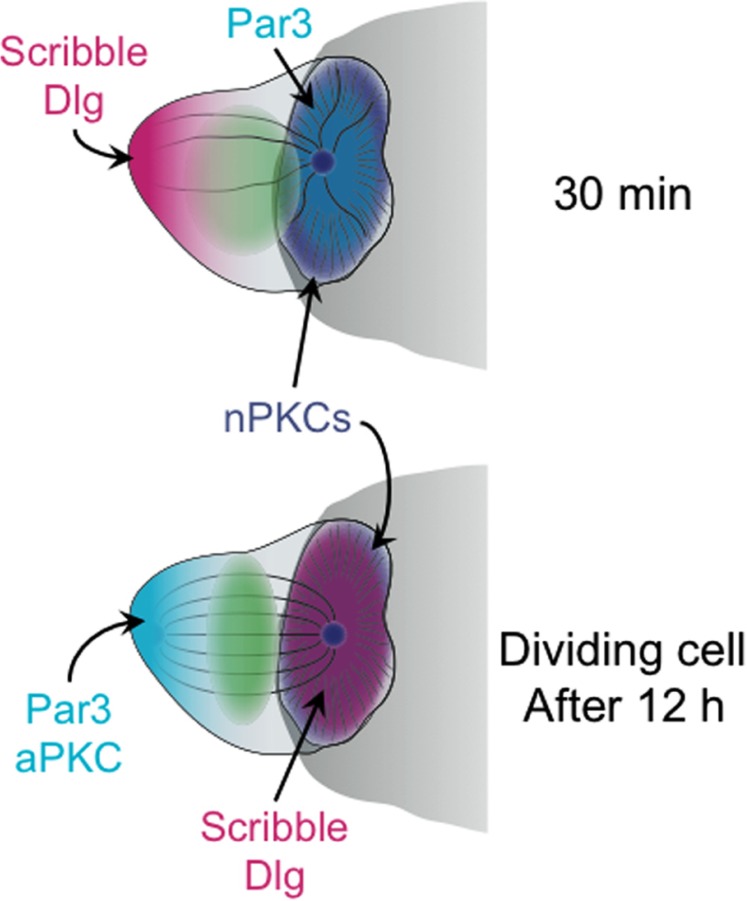
**Schematic diagram showing the distribution of polarity complexes in synaptic T cells 30 min (top), and 12 h (bottom) after antigen recognition**. The cell at 12 h is depicted as undergoing mitosis. The APC is shown as a gray semicircle.

The importance of these complexes for polarity induction in other systems suggested that they might play a similar role in T cells during IS formation. Consistent with this notion, immunocytochemical studies demonstrated that PAR3 accumulates at the T cell–APC interface while Scribble–Dlg localizes to the back of the cell (Ludford-Menting et al., 2005; **Figure 2**). Although the overall distribution of PKCζ was not polarized under these conditions, the phosphorylated and activated form of PKCζ was enriched together with PAR3 at the IS (Bertrand et al., 2010). Blocking this pool of active PKCζ either by application of a small molecule inhibitor or by overexpression of a dominant-negative PKCζ construct disrupted MTOC polarization toward the APC (Bertrand et al., 2010). Polarization responses were also impaired by overexpression of a dominant-negative version of the kinase Par1b (Lin et al., 2009), which is known to antagonize PAR3–PAR6–aPKC in other systems. Although it is somewhat worrying that these perturbation studies exclusively used pharmacological and overexpression approaches, when taken together the results support a role for the PAR complex and aPKC in synaptic T cell polarity.

Notably, the synaptic accumulation of PAR3 and phospho-PKCζ, which was coupled with Scribble–Dlg localization at the back of the cell, was only observed at relatively late time points, 20–30 min after T cell–APC conjugate formation (Ludford-Menting et al., 2005; Bertrand et al., 2010). Similarly, the perturbation experiments documenting the effects of PKCζ inhibition and overexpression were all scored after 20 min or more (Bertrand et al., 2010). This is well after initial MTOC reorientation, which occurs in less than 5 min. Hence, PAR3–PAR6–aPKC may not be required for the act of polarization, but rather for the subsequent stabilization of the polarized state. This interpretation is consistent with recent live-imaging analysis of B cell polarization (Yuseff et al., 2011). In this study, shRNA-mediated suppression of PKCζ did not block initial MTOC reorientation toward antigen coated beads. It did, however, impair the maintenance of polarization over time.

On balance, the extant data are consistent with a model that divides MTOC polarization into two phases: a fast direction sensing and reorientation phase directed by DAG and nPKCs, followed by a stabilization and consolidation phase that requires the PAR3–PAR6–aPKC and Scribble–Dlg polarity complexes. The separation of polarization responses into two distinct steps would presumably facilitate the modulation of synaptic strength and stability, allowing T cells to tailor their synapses for specific biological functions. Fast, transient MTOC reorientation without subsequent synaptic consolidation would presumably be optimal for cytotoxic T lymphocytes (CTLs), which are most effective when they kill quickly and move on. By contrast, long-lived, stable synapses may be crucial for the directional secretion of cytokines by helper T cells, which occurs hours after initial TCR stimulation. Stabilization may also play a key role in properly aligning naïve T cells to undergo asymmetric cell division on the surface of a dendritic cell (DC; see below). Now that some of the molecules controlling the distinct phases of T cell polarity have come to light, it may be possible to test these predictions in physiologically relevant settings.

Interestingly, PAR3–PAR6–aPKC and Scribble–Dlg also appear to be involved in the maintenance of migratory cell polarity. They display asymmetric localization in migrating T cells, with PAR components in the cell body and Scribble–Dlg in the uropod (Ludford-Menting et al., 2005; Real et al., 2007). Furthermore, overexpression of dominant-negative PKCζ leads to cell rounding, as does siRNA knockdown of Scribble (Ludford-Menting et al., 2005; Real et al., 2007). In light of the model for synaptic polarization described above, it is tempting to speculate that migratory polarity may also be established in two steps. Thus, initial direction sensing and leading edge formation would be driven by PIP3 and regulators of Rho-family GTPases, such as the exchange factor Dock2 (Ward and Marelli-Berg, 2009). This would be followed by the formation of polarity complexes and the stabilization of extended, hand-mirror morphology. Time-resolved studies of polarity induction in response to migratory stimuli, such as chemokines, will be required to examine this hypothesis in more detail.

## SYNAPTIC POLARIZATION AND DIRECTIONAL SECRETION

Evidence supporting a role for the IS in directional secretion predates the term “immunological synapse.” Pioneering imaging studies of T cell–APC conjugates, performed by Kupfer et al. (1991), 1994, demonstrated that intracellular compartments containing nascent cytokines were tightly apposed to the cell–cell interface, suggestive of targeted release toward the APC. Similarly, by activating T cells that had been forced into membrane pores, Janeway and colleagues provided evidence for preferential cytokine secretion in the direction of TCR stimulation (Poo et al., 1988). It was subsequently found that T cells use at least two directionally distinct pathways for cytokine secretion, one that targets the IS, and another that releases factors in an unpolarized manner (Huse et al., 2006). Although it remains unclear precisely how different cytokines are targeted to different secretory pathways, cell biological analyses have indicated that these pathways are molecularly distinct (Huse et al., 2006). Thus, synaptically secreted cytokines such as IL-2 and IFNγ were observed to traffic in intracellular compartments coated with the GTPases Rab3d and Rab19. Conversely, multidirectionally released factors such as TNF and the chemokine CCL3 occupied vesicles containing the SNARE (soluble *N*-ethylmaleimide-sensitive factor accessory protein receptor) proteins syntaxin-6 and Vti1b. SNAREs and Rabs have been implicated in vesicular trafficking and compartmental specification in numerous systems (Pfeffer, 2001; Jahn et al., 2003), and it is likely they play a central role in the spatial regulation of cytokine secretion from T cells.

**FIGURE 3:**
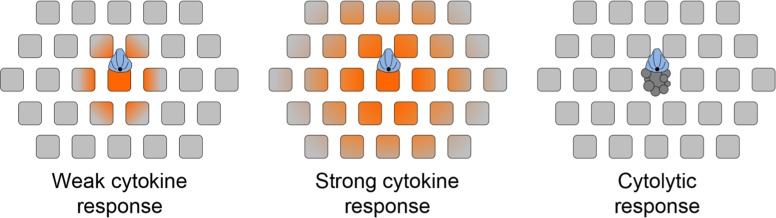
**chematic diagram depicting the effects of synaptic secretory responses**. **(A)** Weak cytokine secretion affects only the APC and cells in its immediate vicinity. **(B)** Strong cytokine secretion affects a larger number of bystander cells. **(C)** Cytolytic killing, mediated by perforin and granzyme, is restricted to the APC. The scope of cytokine diffusion is denoted in orange. T cells are colored blue, with the MTOC and microtubules in black. Bystander cells are depicted as gray squares. Adapted from Sanderson et al. (2012).

The best-studied directional secretion phenomenon in lymphocytes is the synaptic release of cytolytic factors, such as perforin and granzyme, by CTLs and NK cells (Stinchcombe and Griffiths, 2007). Cytotoxic molecules are stored in secretory lysosomes called lytic granules, which are molecularly distinct from compartments involved in cytokine trafficking (Reefman et al., 2010). TCR signaling induces the dynein-dependent trafficking of these granules along microtubules toward the MTOC (Mentlik et al., 2010; Daniele et al., 2011), which concurrently reorients to the IS. In this manner, lytic granules are delivered to the center of the IS, where they fuse with the plasma membrane in a designated “secretory domain” (Stinchcombe et al., 2001; **Figure 3**). Compared to the dense, orthogonal array of actin found in the peripheral lamellipodium, the cortical actin in the secretory domain is sparse and weblike (Brown et al., 2011; Rak et al., 2011). The transient gaps that appear in the actin meshwork in this region provide avenues for the egress of lytic granules and probably also cytokine compartments.

Over the past 10 years, our knowledge of how lytic granules traffic toward the MTOC and the IS has improved substantially. The focusing of lytic granules at the MTOC occurs within minutes of TCR stimulation, and is strongly dependent on Ca^2+^ (Beal et al., 2009). Indeed, the speed of granule trafficking on microtubules scales proportionately with intracellular Ca^2+^ concentration. Notably, both Ca^2+^ influx and DAG accumulation result from the cleavage of PIP2 by PLCγ. Thus, both the reorientation of the MTOC and the focusing of lytic granules respond to the same TCR proximal signaling event, which no doubt facilitates the close temporal coupling of the two responses.

The analysis of proteins associated with lytic granules has revealed other components involved in their trafficking to the MTOC. Particularly relevant to the scope of this review is PKCδ, which binds constitutively to lytic granules and is required for their focusing around the MTOC (Ma et al., 2007, 2008). Importantly, PKCδ deficiency has no effect on MTOC reorientation to the IS, indicating that PKCδ has a specific role in granule trafficking. Recent studies have also implicated the GTPase Rab7 and its associated effector RILP in lytic granule motility (Daniele et al., 2011). The Rab7–RILP complex is involved in coupling multiple distinct organelles to dynein, leading to their accumulation at microtubule minus ends (Wang et al., 2011). In CTLs, both Rab7 and RILP associate with lytic granules, and overexpression of RILP drives copious accumulation of dynein on granule membranes (Daniele et al., 2011). Consistent with a role for this complex in granule trafficking, siRNA knockdown of Rab7 inhibits cytotoxicity. Given the similarities in localization pattern and phenotype, one might imagine that Rab7–RILP and PKCδ cooperate in the same pathway. This remains to be explored, however, as does the relationship between each of these factors and Ca^2+^.

It is generally thought that synaptic secretion by T cells enables selective communication with or killing of APCs in dense intracellular environments packed with bystander cells (Huse et al., 2008). Consistent with this notion, a considerable amount of *in vitro* and *in vivo* work has established that cytotoxic killing is both contact mediated and antigen specific (Kuppers and Henney, 1977; Kupfer et al., 1986; Lanzavecchia, 1986; Breart et al., 2008; Sanderson et al., 2012). Studies have also documented that APCs in direct contact with CD4^+^ T cells are preferentially activated. For example, sustained polarization of the T cell MTOC toward antigen bearing DCs is required to induce strong IL-12 production by those DCs (Bertrand et al., 2010; Tourret et al., 2010). Similarly, B cells that are directly conjugated with helper T cells have been observed to divide before other B cells in the culture (Kupfer et al., 1994). It is unclear, however, whether these preferential effects on synaptically engaged APCs depend on directional cytokine secretion *per se*. Indeed, two recent reports have indicated that IFNγ can diffuse away from the IS and stimulate cells at a great distance from the T cell–APC conjugate. In one study (Sanderson et al., 2012), a fluorescently labeled Stat1 reporter was used to monitor the scope of IFNγ secretion from CTLs *in vitro*. In the other study (Muller et al., 2012), IFNγ-activated macrophages were visualized *in vivo* by staining for iNOS. In both of these systems, the most intense responses to IFNγ were observed in the APC or close to it, consistent with the idea that the APC has privileged access to synaptically secreted cytokines. Nevertheless, it is clear that the IS does not act as an impenetrable barrier to diffusion.

In light of this new data, it is worth reevaluating how directional secretion into the IS might maintain the specificity of secretory responses. Because T cell and APC membranes are closely apposed in the IS, it is likely that the APC would have the first opportunity to bind synaptically secreted factors. This could substantially affect the scope of the secretory response in circumstances where the number of secreted molecules is comparable to the number of cell surface receptors present in the IS. Of course, stronger responses would overwhelm the available receptors on the APC, leading to diffusion out of the IS and the stimulation of bystander cells (**Figure 3**). A prediction of this “first dibs” mechanism is that small differences in the magnitude of cytokine production could have very substantial effects on the scope of the subsequent response. The extent of bystander effects probably also depends on the physical properties of the secreted factors themselves. Cytokines are small, stable proteins, and they diffuse quickly in tissue environments (G. Altan-Bonnet, personal communication). By contrast, perforins are unstable at neutral pH and readily oligomerize to form pores in cellular membranes (Pipkin and Lieberman, 2007). Indeed, it is likely that secreted perforins have only a short lifetime as soluble factors, associating quickly with the opposing APC. Thus, the specificity of killing would be maintained by the relative instability of perforin, and not by a diffusion barrier imposed by the IS. This model is consistent with the observation that CTLs mediate highly specific killing even while inducing IFNγ signaling responses in distal bystanders (**Figure 3**; Sanderson et al., 2012). Finally, it is possible that specificity is achieved by the combinatorial action of soluble and membrane bound signals. For example, full activation of B cells and DCs requires both cytokine signaling as well as engagement of the cell surface receptor CD40 (Foy et al., 1996; Snijders et al., 1998). CD40 binds to the transmembrane ligand CD154, which accumulates in the T cell IS in response to TCR stimulation (Boisvert et al., 2004; Bertrand et al., 2010; Tourret et al., 2010). The synaptic localization of CD154 provides preferential access to the APC, and would presumably limit the scope of full activation even under conditions where stimulatory cytokine is freely available. An analogous mechanism may also operate during cytotoxic responses, which often require Fas–FasL interactions in addition to perforin- and granzyme-mediated effects (Russell and Ley, 2002).

It is becoming increasingly clear that synaptic secretion, on its own, does not constrain responses to the APC. Identifying the mechanisms or combinations of mechanisms that complement synaptic secretion in order to maintain specificity should provide a more nuanced (and accurate) view of how T cells shape the scope of their effector responses.

## POLARITY CUES AND ASYMMETRIC CELL DIVISION

One of the most exciting developments in lymphocyte polarity over the past 5 years is the discovery that antigen-stimulated T cells undergo asymmetric cell division (Chang et al., 2007). Certain proteins, including CD8 and LFA-1, were found to preferentially accumulate on one side of mitotic T cells, implying that they might be inherited unequally by daughter cells. Consistent with this hypothesis, flow cytometric analyses of proliferated T cells revealed a bimodal distribution of these markers. Asymmetric division was only observed under conditions of infection, suggesting that it might play a role in the differentiation of effector cells. Indeed, it was found that daughter cells expressing lower levels of CD8 were more likely to develop into memory cells, while daughters expressing high levels of CD8 became effectors. Hence, asymmetric cell division is likely to play a significant role in the development of distinct T cell subsets after infection. Recently, it was found that activated B cells also undergo asymmetric division (Wang et al., 2011), indicating that this may be a general differentiation mechanism common to all lymphocytes.

The PAR3–PAR6–aPKC and Scribble–Dlg complexes are important for asymmetric division in multiple cell types (Nance and Zallen, 2011). Because previous studies had shown that these proteins accumulate asymmetrically in synaptic T cells (Ludford-Menting et al., 2005), their role in subsequent cell division was investigated. Indeed, perturbation of both complexes inhibited asymmetric division, and disruption of the Par6–PKCζ interaction with a small molecule inhibitor impaired memory cell development *in vitro* (Oliaro et al., 2010). Consistent with these observations, it was found that PAR3, PKCζ, Scribble, and Dlg remained polarized in synaptic T cells at 10–40 h after antigen recognition (Oliaro et al., 2010), the time period during which cell proliferation occurs. However, this polarization pattern was a complete reversal of the one observed at 30 min. After 10 h, Scribble–Dlg became enriched in the IS, while PAR3 and PKCζ localized to the back of the cell (**Figure 2**). When and how this remarkable inversion of polarity occurred is not known. It is also unclear why inverting the localization of polarity complexes should be necessary for promoting asymmetric division. In that regard, it is intriguing to note that PKCθ (and perhaps other nPKCs, as well) remained localized to the IS even at this late stage (Oliaro et al., 2010). Perhaps, the axis of PAR3–PAR6–aPKC/Scribble–Dlg polarity must be reversed relative to the axis of DAG/nPKC polarity in order for productive division to occur? The role of MTOC polarization during asymmetric cell division is also mysterious. One might expect that the placement of the MTOC at the IS could dictate the orientation of spindle assembly. However, imaging of late stage synapses indicated that there is a relaxation of MTOC polarization prior to mitosis (Oliaro et al., 2010). Whether the residual nPKC activity at the IS serves to repolarize the spindle once it forms awaits further studies. There is clearly a need for more mechanistic work in this area.

## CONCLUDING REMARKS

Although our understanding of both the mechanisms that drive synaptic polarity in lymphocytes and the effector responses facilitated by that polarization has improved in recent years, many important questions remain. It is now clear that nPKCs and aPKCs play important roles in promoting MTOC polarization. Precisely, how they control dynein and other cytoskeletal components during this process, however, is unknown. To address this question, it will be crucial to identify the PKC substrates and interacting proteins relevant to this system.

It is also unclear how T cells generate and maintain such a stable accumulation of DAG at the IS. The answer likely involves the highly coordinated regulation of DAG production and destruction. In that regard, we have found that pharmacological inhibition of DGKs, which play a major role in the metabolism of DAG in T cells (Zhong et al., 2008), results in profound destabilization of synaptic DAG accumulation and impaired MTOC reorientation (Quann et al., 2009). T cells express multiple DGKs, and determining which isoform(s) contribute to T cell polarity will be an important challenge.

The concept that MTOC polarization has initiation and stabilization phases is quite intriguing, and requires further investigation. It will be particularly interesting to determine how and when activated T cells transition from one phase to the next. It is conceivable that polarity complexes are recruited to the IS by DAG and nPKC signaling. However, it is also possible that the MTOC itself triggers the requisite signaling events once it is positioned at the IS. A large number of unique signaling proteins are associated with the MTOC, and the close apposition of these proteins with plasma membrane components at the IS could profoundly affect local signaling.

The link between MTOC polarization and directional secretion is well established, and the indications it is important for asymmetric cell division are encouraging. However, it is quite likely that there are other downstream responses requiring polarization of the MTOC to the IS that will remain unknown until we can selectively disrupt the process *in vivo*. Knowledge of the molecular mechanisms controlling cytoskeletal polarization in lymphocytes should, in future years, enable investigators to achieve this overarching goal.

## Conflict of Interest Statement

The author declares that the research was conducted in the absence of any commercial or financial relationships that could be construed as a potential conflict of interest.
